# Data-driven vestibular fMRI in PPPD and residual dizziness after BPPV: a narrative methodological review of AI-based phenotyping and longitudinal modeling

**DOI:** 10.3389/fneur.2026.1915520

**Published:** 2026-09-23

**Authors:** Bingxing Yang, Liqiong Zhou, Siqi Wang, Yi Du, Peng Zhang, Suwen Bai, Zhaoxia Wang, Fang Ma, Guihao Guan, Xi Chen, Juanjuan Li, Xianhai Zeng

**Affiliations:** 1 Department of Graduate and Scientific Research, Zunyi Medical University Zhuhai Campus, Zhuhai, Guangdong, China; 2 Department of Otolaryngology, Longgang Otolaryngology Hospital & Shenzhen Key Laboratory of Otolaryngology, Shenzhen Institute of Otolaryngology, Shenzhen, Guangdong, China; 3 Senior Department of Otolaryngology Head and Neck Surgery, Chinese PLA General Hospital, Beijing, China; 4 State Key Laboratory of Hearing and Balance Science, Beijing, China; 5 National Clinical Research Center for Otolaryngologic Diseases, Beijing, China

**Keywords:** artificial intelligence, benign paroxysmal positional vertigo, brain network, dizziness, functional magnetic resonance imaging, persistent postural-perceptual dizziness, statistical modeling

## Abstract

Neuroimaging studies of vertigo and chronic dizziness have identified abnormalities across vestibular, visual, somatosensory, and cognitive-emotional networks, but conventional group-level analyses are limited in capturing individual heterogeneity and longitudinal change. This narrative methodological review integrates current evidence and proposes a framework combining functional magnetic resonance imaging (fMRI), artificial intelligence (AI), and advanced statistical modeling, anchored in two priority research scenarios: PPPD phenotyping and longitudinal characterization of residual dizziness after BPPV repositioning. AI, particularly unsupervised learning, may help identify candidate network subtypes from high-dimensional fMRI features, whereas mixed-effects, nonlinear, and latent trajectory models can evaluate their longitudinal stability, clinical relevance, and temporal evolution. Together, these approaches form a complementary pattern-discovery–longitudinal-validation framework. Current evidence, however, remains limited by small samples, single-center designs, technical heterogeneity, and scarce external validation. Accordingly, candidate PPPD subgroups and proposed recovery trajectories after BPPV repositioning should be regarded as hypothesis-generating rather than established fMRI phenotypes. The framework is therefore intended primarily as a research methodology for mechanistic investigation, candidate biomarker discovery, and prospective validation rather than as a clinically deployable decision tool. Future progress will require larger multicenter cohorts, harmonized acquisition and preprocessing, rigorous control of confounding and multiple testing, independent validation, and demonstration of incremental value beyond established clinical assessment.

## 1 Introduction

Vertigo is a common clinical syndrome characterized primarily by spatial disorientation; it is highly clinically heterogeneous and imposes a significant burden on public health. Epidemiological data indicate that neuroimaging studies (including head CT and brain MRI) are widely used among patients presenting with dizziness in outpatient and emergency settings. Brain MRI accounts for 25% of all imaging studies and constitutes a major component of healthcare expenditures (1). More importantly, patients with peripheral vertigo had a significantly higher risk of depression (hazard ratio HR = 2.91) and anxiety (HR = 4.92) within 2 years after discharge, highlighting its profound impact on mental health and quality of life (2). The causes of vertigo are complex and encompass a variety of conditions, including vestibular migraine (VM), Meniere’s disease (MD), persistent postural-perceptual dizziness (PPPD), vestibular neuritis (VN), and benign paroxysmal positional vertigo (BPPV). Because their clinical presentations overlap and diagnostic criteria vary, this leads to high rates of misdiagnosis and significant variability in treatment response (3). For example, VM and MD can be manifested as paroxysmal vertigo, but the distribution pattern of endolymphatic hydrops (EH) in VM and MD is quite different: 32% of VM patients have bilateral, low-grade, vestibular EH, while 80% of MD patients have unilateral, high-grade, cochlear EH (4). This difference in pathophysiological mechanisms highlights the limitations of symptom-based classification alone and motivates the search for objective, quantifiable neuroimaging markers that may improve mechanistic characterization and, after appropriate validation, potentially contribute to more precise clinical stratification.

Functional magnetic resonance imaging (fMRI) detects indirect changes in neuronal activity through blood oxygen level-dependent (BOLD) signals, enabling the non-invasive mapping of the dynamic characteristics of whole-brain functional networks (5). fMRI not only identifies the distributed network comprising cortical, subcortical, cerebellar, and brainstem regions associated with vestibular function, but also reveals the patterns of integration between these regions and the cognitive, emotional, and somatosensory systems (6). Resting-state fMRI has been used to characterize the spatio-temporal dynamic abnormalities of brain function in patients with vertigo. For example, the ALFF and ReHo dynamics of patients with vestibular migraine are increased in bilateral paracentral lobules and supplementary motor areas, while decreased in right temporal pole, posterior cerebellar lobe and angular gyrus, and the ALFF dynamics of left middle occipital gyrus is positively correlated with the course of vertigo (7). Task-based fMRI can induce activation of specific brain regions by simulating vestibular stimulation (such as virtual roller coaster task), revealing the dysfunction of vestibular-visual integration pathway (8). It is worth noting that conducting fMRI without anesthesia provides a more accurate reflection of neurophysiological states and avoids interference from anesthesia on cerebrospinal fluid flow and neurovascular coupling (9). In addition, fMRI can be fused with other modalities (such as arterial spin labeling and structural MRI) to construct multidimensional neuroimaging models, thereby improving the ability to classify complex diseases (10, 11). Despite these advances, traditional neuroimaging analysis still faces numerous challenges. Most studies are cross-sectional and have small sample sizes (12); imaging features are often high-dimensional and highly correlated (for example, in functional connectivity studies, researchers must handle correlation matrices involving hundreds to thousands of brain regions) (13); clinical phenotypes are heterogeneous; and, more critically, several large-scale functional MRI studies have shown that the stability of brain networks declines along a nonlinear trajectory throughout the lifespan (14). These limitations (cross-sectional small samples, high-dimensional collinear features, clinical heterogeneity, and non-linear brain network changes) mean that traditional analysis alone cannot satisfy both robustness and interpretability. These limitations motivate the use of data-driven approaches capable of handling high-dimensional collinearity, individual heterogeneity, and nonlinear dynamics. Artificial intelligence and advanced statistical modeling offer complementary solutions. First, the former excels at identifying multivariate imaging patterns from high-dimensional, multicollinear data for classification or stratification purposes (15, 16), while the latter can account for multicenter structures, repeated measures, individual heterogeneity, and dynamic compensation through mixed-effects, nonlinear, and trajectory models (17, 18). The purpose of this narrative review is to critically synthesize relevant evidence and propose a methodological framework integrating fMRI network analysis, artificial intelligence, and advanced statistical modeling for hypothesis generation, biomarker discovery, and future validation in vestibular research.

Rather than serving as a clinically deployable decision tool, this framework is intended to provide a research strategy for investigating clinically motivated questions that remain difficult to resolve using conventional assessments. In neurology and otolaryngology outpatient clinics, two clinical scenarios in particular highlight the limitations of the existing diagnostic framework. PPPD is a functional vestibular disorder defined by symptomatic criteria. Existing classification studies suggest that the condition exhibits neurobiological heterogeneity and can be divided into different subtypes (19); clinically, there are significant differences in vestibular rehabilitation responses among patients diagnosed with PPPD, which provides clinical evidence supporting the existence of subtypes within the unified diagnosis of PPPD (20); Even after successful otolith repositioning, a significant proportion of patients with benign paroxysmal positional vertigo continue to experience residual dizziness; the underlying mechanism goes beyond simple peripheral otolith disorders, with the core issue pointing to individual differences in central vestibular compensatory capacity (21). When traditional clinical symptomatology and vestibular function tests cannot provide sufficient resolution, can objective markers at the brain network level provide incremental value? This is the core issue that the methodological framework reviewed in this review attempts to address. As shown in Figure 1, the framework starts with vestibular network and fMRI-based image data, and is verified by artificial intelligence-based classification, stratification and statistical verification. Ultimately, this framework aims to generate and validate candidate imaging phenotypes and longitudinal markers that may, if sufficiently validated, inform future studies of diagnosis, prognosis, and treatment stratification. Rather than treating PPPD and residual dizziness following BPPV repositioning as two parallel examples, this review uses them at different levels of a peripheral-to-central hierarchy of vestibular dysfunction and compensation. Residual dizziness after successful BPPV repositioning follows a well-defined peripheral vestibular disorder and therefore provides a useful setting in which to investigate whether central sensory recalibration and compensation remain incomplete after correction of the peripheral trigger. PPPD, by contrast, is characterized predominantly by persistent disturbances in perceptual weighting, visual dependence, attentional processing, and multisensory integration, thus representing the higher-order end of this hierarchy for investigating heterogeneity in higher-order vestibular network organization. This axis is conceptual rather than a proposed temporal progression from BPPV to PPPD and is used here to organize different research questions across levels of vestibular processing.

**Figure 1:**
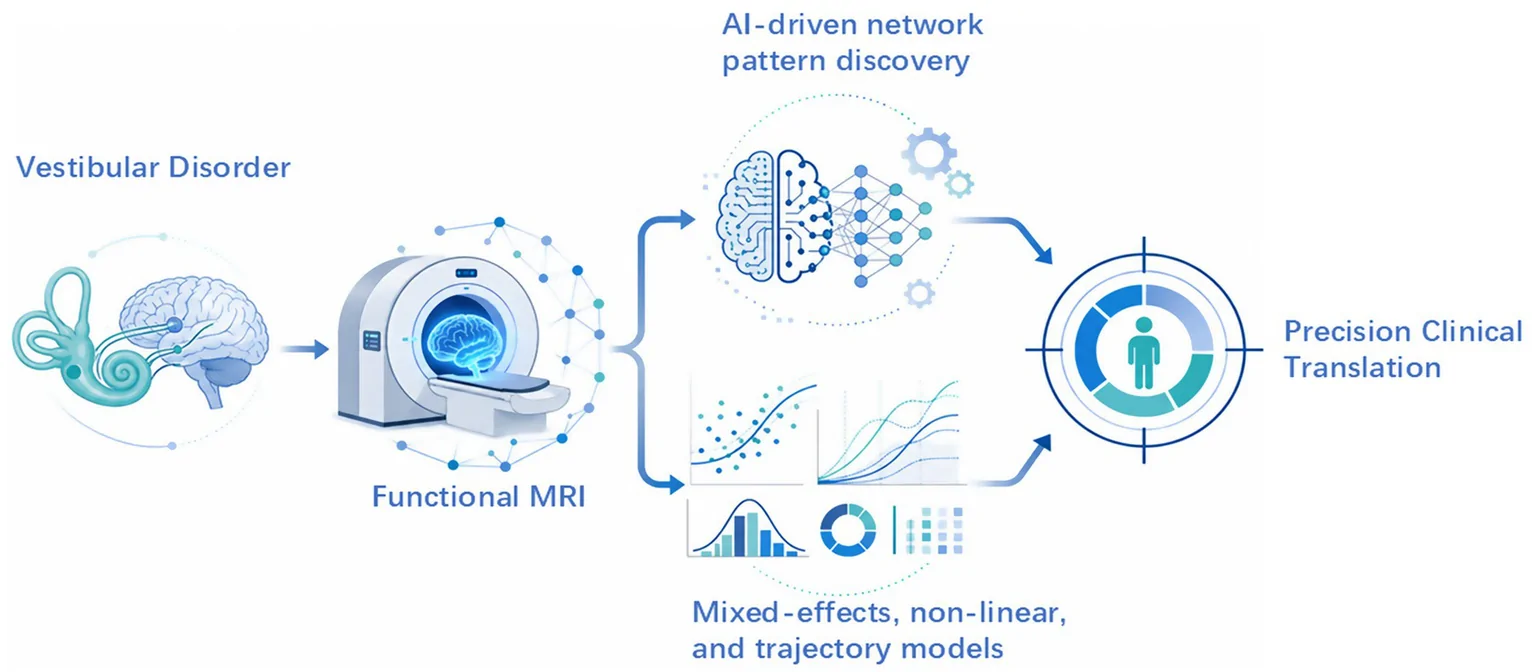
An integrated analytical framework for mechanistic investigation and future clinical translation in vestibular neuroimaging.

## 2 Literature search and evidence selection

This article was designed as a structured narrative methodological review rather than a systematic review or meta-analysis. Accordingly, no formal systematic-review protocol or predefined inclusion/exclusion criteria were applied. Literature selection was guided by the methodological questions addressed in this review and was updated iteratively during manuscript development and revision.

PubMed was the primary database. No lower publication-date limit was imposed, as the methodological scope of this review required consideration of both foundational studies establishing current concepts of vestibular cortical and network organization and more recent developments in fMRI, artificial intelligence, and longitudinal modeling. The search was last updated in August 2026. Search concepts included combinations of terms related to “vestibular,” “vertigo,” “dizziness,” “PPPD,” “BPPV,” “residual dizziness,” “fMRI,” “functional connectivity,” “machine learning,” “artificial intelligence,” “mixed-effects models,” “nonlinear models,” and “trajectory modeling.” For methodological and computational topics that were not readily retrievable through PubMed, supplementary searches were conducted using arXiv and targeted title-based web searches. When both preprint and formally published versions were available, the peer-reviewed or conference-published version was preferentially cited.

Evidence selection focused on studies relevant to four areas: (1) vestibular-related fMRI networks and network alterations in vestibular disorders; (2) AI or machine-learning approaches relevant to classification, prediction, or data-driven phenotyping; (3) methodological or empirical studies informing mixed-effects, nonlinear, and latent trajectory analyses; and (4) evidence directly relevant to PPPD phenotyping and residual dizziness following BPPV repositioning. During revision, additional targeted searches were conducted to address methodological issues raised during peer review, including multicenter harmonization, sample-size and power considerations, explainable AI, psychiatric confounding, and reproducibility of data-driven subtyping.

Because direct evidence combining vestibular fMRI with AI or advanced longitudinal modeling remains limited, evidence from adjacent neuroimaging, psychiatric, clinical, and statistical fields was included when it provided a clear methodological precedent. Throughout the review, these methodological precedents are distinguished from direct empirical evidence in vestibular fMRI and from hypotheses proposed for future investigation.

## 3 Vestibular-related brain networks and the foundations of fMRI research

### 3.1 The human vestibular cortical network

The human vestibular cortical network is a highly distributed, multilevel functional system that encompasses multiple key regions, including the cortex, subcortical structures, cerebellum, and brainstem, which together form the central hub for processing vestibular information (6). The network is not only involved in spatial orientation and balance regulation, but also closely related to advanced brain functions such as cognition, emotion and body representation (6). At the cortical level, the regions involved in vestibular signal processing primarily include the paracentral sulcus (areas 2v and 3aV), frontal area 6, areas 7a and 7b of the supramarginal and marginal gyri, and the parietal-insular vestibular cortex (PIVC) located within the lateral fissure. The PIVC maintains dense bidirectional neural connections with the vestibular nuclei and all of the aforementioned cortical regions (22–26). Recent studies have shown (27) that the distribution of vestibular signals in the cortex is far more extensive than previously thought within the classical framework; rather than being confined to a single region, these signals extend to the primary visual cortex, the posterior parietal cortex, the inferior parietal gyrus, and the post-olfactory cortex, among others, collectively forming a distributed cortical network for multisensory integration. Each of these regions can respond independently to vestibular stimuli while simultaneously integrating visual and somatosensory information to support proprioceptive awareness and spatial navigation. In terms of connectivity patterns, the core vestibular network exhibits extensive connections—both structurally (28) and functionally (29)—with brain regions involved in visuospatial processing, arousal and attention regulation [e.g., the anterior insula (30–32)], sensory gain modulation, proprioception [superior temporal gyrus (33, 34)], sensorimotor processing [superior temporal gyrus and cingulate cortex (35, 36)], cognitive-perceptual (37), and emotional processing [superior temporal gyrus, subgenual cortex, and anterior insula (38–42)]. The thalamus acts as the relay-integration hub of the entire network (43), and its upstream and downstream connect the cortical signals with the cerebellum’s motion correction system (44) to jointly achieve reflex control of posture and conscious motion perception.

### 3.2 Common fMRI technical approaches in vestibular research

At present, the technology has developed a variety of technical routes suitable for vestibular research. Resting-state fMRI (rs-fMRI) can evaluate the spatial and temporal characteristics of spontaneous neural activity without task stimulation. Commonly used indicators include regional homogeneity (ReHo), amplitude of low frequency fluctuation (ALFF) and its fractional form (fALFF). These parameters have been used to identify abnormal brain activity patterns in VM and PPPD patients (7, 45). Task-state fMRI induces specific neural responses by applying vestibular stimulation (such as low-frequency sinusoidal current vestibular stimulation, sGVS) to locate functional areas involved in vestibular processing. For example, sGVS combined with transcranial alternating current stimulation (tACS) can significantly change the BOLD signal of the anterior parietal-insular vestibule cortex, thalamus and brainstem nuclei (the following olivary nucleus, solitary tract nucleus), revealing the top-down regulatory pathway (46). In addition, functional connectivity analysis (including static and dynamic connectivity) is widely used to characterize the synergy within the vestibular network and with other brain networks (such as sensorimotor networks, visual networks) (7, 45).

### 3.3 Vestibular-specific technical considerations for fMRI acquisition and preprocessing

Vestibular fMRI requires particular attention to head motion, scan tolerability, medication exposure, and the standardization of vestibular stimulation paradigms. Even small head movements can introduce systematic bias into resting-state functional-connectivity estimates (47), and this issue is especially relevant when symptomatic participants experience dizziness, nausea, or difficulty maintaining a stable head position during scanning. Motion-control procedures should therefore be prespecified and should distinguish between subject-level exclusion criteria and volume-level censoring based on framewise displacement or related measures. Studies should additionally report the amount of data removed during censoring and examine whether residual motion differs systematically between groups. As a practical example, a recent longitudinal resting-state fMRI study of BPPV patients with residual dizziness used functional outlier detection incorporating a 0.9-mm subject-motion threshold and subsequently excluded several participants because of excessive head motion or poor spatial normalization (48). Such examples illustrate that motion-related data loss may be nontrivial in vestibular cohorts and should be incorporated into both quality-control reporting and prospective sample-size planning.

Beyond censoring or exclusion, data-driven denoising approaches can help reduce motion-related artifacts. ICA-AROMA, for example, decomposes single-subject fMRI data into independent components, identifies components likely to reflect motion using predefined spatial and temporal features, and removes their contribution through regression (48). This approach can complement conventional realignment and censoring strategies, although no single motion-control procedure or threshold is universally optimal. The selected strategy should therefore be defined prospectively and applied consistently across groups.

Scan duration represents a second practical trade-off between patient tolerability and measurement reliability. Prolonged immobility may be difficult for participants with active dizziness or poor tolerance of the scanner environment. Relatively concise acquisitions are feasible in vestibular cohorts; for example, the longitudinal BPPV residual-dizziness study described above acquired resting-state fMRI for 7 min, yielding 210 time points per participant (48). However, general resting-state fMRI evidence indicates that excessively short acquisitions reduce the reliability of functional-connectivity estimates (49). Protocols should therefore prioritize the prespecified primary functional acquisition and essential structural sequences, while shorter or segmented acquisitions may be considered when necessary for participants with poor tolerance or claustrophobic discomfort. The duration of usable data after quality control should be reported explicitly.

Medication exposure is another potential confounder. Vestibular medications and psychotropic treatments may influence BOLD-derived activity or connectivity independently of vestibular pathology. For example, longitudinal pharmacological fMRI studies have demonstrated changes in resting-state brain activity following SSRI administration (50), whereas direct evidence regarding the effects of commonly used vestibular medications such as betahistine on vestibular BOLD signals remains limited. Medication type, dose, treatment duration, and timing relative to scanning should therefore be documented and considered in stratified or sensitivity analyses where appropriate.

Finally, resting-state and task-based fMRI provide complementary information but impose different standardization requirements. Resting-state acquisition is generally easier to harmonize across repeated or multicenter studies, whereas task-based vestibular paradigms require additional control because the stimulation itself may provoke dizziness, discomfort, or motion. Galvanic and caloric vestibular stimulation have both been implemented during fMRI (51, 52), while visual self-motion paradigms have also been used in migraine-related vestibular research (8). For galvanic stimulation, parameters such as electrode montage and polarity, current intensity, waveform or frequency, stimulation timing, perceptual threshold, and scanner compatibility should be standardized and reported. Visual self-motion paradigms should similarly specify stimulus characteristics such as visual field, motion speed, contrast, block duration, fixation conditions, and symptom provocation. Careful documentation of these parameters is important for interpreting between-study heterogeneity and improving reproducibility.

### 3.4 Multicenter harmonization and site effects in vestibular fMRI

Multicenter acquisition will likely be necessary to obtain sufficiently large vestibular fMRI cohorts, but differences in scanner manufacturer, hardware, field strength, acquisition parameters, and preprocessing can introduce substantial non-biological variability into functional-connectivity measures. Recent traveling-subject studies have demonstrated limited subject-level reproducibility of functional connectivity across scanner vendors, with post-hoc harmonization reducing scanner-related variance more effectively at the group than at the individual level (53). Prospective harmonization of acquisition and reconstruction may therefore be preferable to relying solely on statistical correction after data collection (54).

These issues are particularly relevant to vestibular neuroimaging because networks of interest frequently include small brainstem and cerebellar structures. High-resolution brainstem fMRI requires careful spatial normalization and physiological-noise correction, and recent 7 T studies have demonstrated the importance of dedicated denoising approaches (55). Moreover, direct comparison of 3T and 7T brainstem functional connectivity has shown stronger cross-field translatability for brainstem–cortical than for brainstem–brainstem connections, indicating that field strength and spatial resolution should be considered explicitly when pooling data involving small vestibular-related structures (56).

Future multicenter studies should therefore harmonize key acquisition and quality-control procedures prospectively, use centralized preprocessing where feasible, and explicitly model residual site/scanner effects. Traveling-subject or phantom measurements can help quantify scanner variability, while statistical harmonization may be considered for derived imaging features. For machine-learning analyses, site-stratified or leave-one-site-out validation should be used whenever feasible to assess cross-site generalizability.

### 3.5 fMRI features and vestibular network phenotype

Based on the above technical route, researchers have characterized a series of abnormal phenotypes of fMRI networks in a variety of vestibular diseases. Although these findings are derived from different diagnostic groups, they show high consistency on several core brain networks.

The Default Mode Network (DMN) is one of the most frequently reported abnormal regions. In PPPD, the functional connectivity between the posterior DMN and the visual network was enhanced, and the degree of enhancement was negatively correlated with the Dizziness Handicap Inventory (DHI) score (57). Patients with VM also exhibit abnormal functional connectivity between the DMN and the visual network and the insular-vestibular cortex; furthermore, dynamic functional network connectivity (dFNC) analysis reveals that patients spend an extended period in states characterized by sensory-attentional coupling, and this duration is positively correlated with scores on the Headache Impact Test-6 (HIT-6) and the DHI (58). In BPPV patients with residual dizziness, it was also observed that the increased functional connectivity between DMN and visual network was positively correlated with the duration of residual dizziness (RD) (59).

The anomalies of the salience network and the edge system constitute the second group of consistency findings. Functional connectivity between the insula, thalamus and anterior cingulate cortex decreased in VM patients (11). In patients with MD, regions such as the parahippocampal gyrus, fusiform gyrus, and supramarginal gyrus exhibit changes characterized by local consistency and low-frequency amplitude, which are significantly correlated with anxiety and depression scores (60). An fMRI experiment with both vestibular stimulation and fear conditioning directly proved that the bilateral insula and prefrontal cortex were the common activation areas of vestibular signal and emotional threat assessment (61).

Abnormalities in the proprioceptive-motor network and the cerebellum reflect compensatory and reweighting processes following vestibular damage. The efficiency of multiple nodes in the sensorimotor network of patients with chronic unilateral vestibular disease decreased, and the functional connectivity of brainstem-sensorimotor network was negatively correlated with DHI score (62). PPPD research has revealed a “vestibular-visual reweighting” pattern—specifically, a selective weakening of connections between the cerebellum and the multimodal vestibular cortex, accompanied by a compensatory strengthening of connections between the cerebellum and the visual network (63).

In addition to localized network abnormalities, changes in whole-brain topological efficiency have also been frequently reported. The gray matter morphological network of VM patients showed a pattern of increased local clustering coefficient and decreased node efficiency in the left superior temporal gyrus (64). Patients with PPPD also exhibit reduced efficiency in information transmission between the visual, sensorimotor, and vestibular cortices, and this impairment in topological efficiency is negatively correlated with DHI scores (45). Chronic unilateral vestibulopathy (CUVP) is characterized by an increase in path length and a simultaneous decrease in both global and local efficiency (62).

An important interpretative challenge is that abnormalities in these networks are not specific to vestibular dysfunction. Anxiety and depression are common in chronic dizziness and are themselves associated with altered activity or connectivity involving the default mode, salience, insular, and anterior cingulate systems (65). This overlap complicates attribution of network abnormalities exclusively to vestibular pathology (65, 66). At the same time, affective symptoms may interact bidirectionally with dizziness and perceptual symptoms, particularly in PPPD, and should therefore not be treated automatically as nuisance variables (65). Future studies should assess anxiety and depression systematically and consider their potential roles as confounders, effect modifiers, or clinically meaningful components of the phenotype according to the research question.

## 4 Artificial intelligence for phenotyping and classification

The above fMRI network phenotypic differences are derived from statistical inference at the population level. They confirm that these network characteristics differ significantly across different diseases, but they cannot directly answer a key clinical question. Considering such a situation: both patients met the diagnostic criteria of PPPD, both showed postural instability and visual intolerance for more than 3 months, and received vestibular rehabilitation training with the same program. However, while one patient showed significant improvement in symptoms after 6 weeks, the other showed no progress. Traditional clinical assessment of symptoms cannot explain this difference in treatment response. In this case, does the diagnostic label ‘PPPD ‘itself mask a very different neurobiological basis? If there is heterogeneity within the label that has not been identified, is there a path to bypass the unified label and directly divide the internal subtypes based on the characteristics of the brain network? This clinically motivated question provides a rationale for using artificial intelligence—particularly unsupervised learning—as a research tool to investigate whether reproducible neurobiological subgroups exist within the current diagnostic label.

### 4.1 Supervised learning in vestibular fMRI: current status and limitations

Machine learning (ML) is most useful when the diagnostic problem is multivariate, pattern-based and cannot be reduced to a single image marker. In fMRI study, supervised learning models can use regional activity, functional connectivity matrices, graph theory metrics, or dynamic network features as output variables to classify patient and control groups or distinguish clinically overlapping syndromes (64, 67–69). However, the current evidence base should be interpreted with caution. Compared with other neurological fields, the direct application of AI in vertigo diagnosis based on fMRI is still relatively rare, and the generalization of AI model is insufficient and biased. At the same time, it also faces concerns about patients ‘data privacy (70). Much of the existing research on machine learning for vertigo relies on clinical vestibular testing, eye movement, or auditory data (71), rather than fMRI. Although resting-state fMRI studies have increasingly identified group-level alterations in PPPD, vestibular migraine, and Ménière’s disease, most remain group-level investigations rather than individual-level machine-learning analyses. Among the studies identified in this review, three published studies directly integrated machine learning with resting-state fMRI-derived functional/effective connectivity or network-related features in vestibular disorders (Table 1), covering diagnostic classification, prediction of disease transition, and differential diagnosis. However, all were single-center studies without independent external validation, indicating that this field remains at an early exploratory stage. Methodological caution is also warranted when interpreting the reported predictive performance. In Lee et al. (57), features used for classification were selected on the basis of group-level differences, a potential source of selection bias acknowledged by the authors. Similarly, in Fu et al. (72), feature selection appears to have preceded the reported cross-validation procedure, raising the possibility of optimistic performance estimates if feature selection was not nested within the training folds.

**Table 1:** Published studies directly integrating machine learning with resting-state fMRI-derived functional connectivity, effective connectivity, or network-related features in vestibular disorders.

Study	Clinical task and sample	rs-fMRI features	ML approach	Validation	Main performance	Methodological considerations
Lee et al., (57) 2018	Diagnostic classification: 38 PPPD vs. 38 HC	Whole-brain functional connectivity; 11 connectivity markers entered into classification	PCA + SVM	LOOCV	Sensitivity 78.4%; specificity 76.9%; accuracy 77.6%; AUC 0.88	Proof-of-concept, single-center study. Feature selection was based on significant group differences before classification, which the authors acknowledged could bias classification results; no dizzy disease-control group.
Fu et al., (72) 2025	Prediction of disease transition: 101 successfully treated BPPV patients; 37 subsequently developed PPPD and 64 did not	ALFF, ReHo, DC and FC; 22 retained features (2 ALFF, 1 ReHo, 1 DC, 18 FC)	Logistic regression, passive-aggressive, LightGBM, SVM, MLP and Gaussian process; SHAP interpretation	5-fold CV	MLP: AUC 0.93; accuracy 0.82; precision 0.83; recall 0.82; F1 0.82	Small single-center cohort; no external validation. Feature reduction from 7,018 to 22 features was performed before the reported model CV, warranting caution regarding possible optimistic performance estimates.
Dai et al., (66)2026	Differential classification: 56 VM, 58 episodic migraine (EM), 63 HC	Bilateral OP2-based effective connectivity derived using voxel-wise Granger causality analysis	Linear SVM; two binary models (VM vs. EM; VM vs. HC)	LOOCV; 1,000 permutation tests; stratified 5-fold CV	VM vs. EM: AUC 0.8264, accuracy 0.7632; VM vs. HC: AUC 0.7764, accuracy 0.6975	No independent external cohort; classification restricted to OP2-related EC measures showing group differences. Authors call for independent multicenter validation.

ALFF, amplitude of low-frequency fluctuation; DC, degree centrality; EC, effective connectivity; EM, episodic migraine; FC, functional connectivity; HC, healthy control; LOOCV, leave-one-out cross-validation; MLP, multilayer perceptron; OP2, parietal operculum cortex 2; PPPD, persistent postural-perceptual dizziness; ReHo, regional homogeneity; SVM, support vector machine; VM, vestibular migraine.

### 4.2 Conventional supervised algorithms: choices and caveats

Among conventional supervised approaches, support vector machines (SVMs) are widely used for high-dimensional neuroimaging classification, although their performance depends on appropriate feature selection and model tuning (73, 74). Random forests (RFs) can capture nonlinear relationships and interactions while providing measures of feature importance, although such importance estimates may be biased or difficult to interpret in the presence of strongly correlated predictors (75). Gradient-boosting methods such as XGBoost can similarly model complex nonlinear relationships and interactions, but careful regularization and validation are required when they are applied to small, high-dimensional cohorts (76). No single algorithm should therefore be assumed to be intrinsically superior for vestibular fMRI. Model comparison should use leakage-free feature selection and appropriate cross-validation procedures, with independent external validation whenever feasible (74, 77–79).

### 4.3 Deep learning: opportunities and challenges

Deep learning extends conventional machine learning by learning hierarchical representations directly from complex imaging data, which is theoretically attractive because fMRI data possess spatial, temporal, and network structure. The suitability of different architectures depends on how fMRI information is represented: convolutional neural networks (CNNs) may be useful when spatial activation patterns, volumetric maps, or connectivity matrices are represented in image-like forms (80–82); graph neural networks (GNNs) are conceptually appealing for functional connectivity analysis because brain regions and their connections can be represented naturally as nodes and edges (83); and transformer-based architectures offer a potential approach for modeling long-range dependencies across time points, brain regions, or dynamic connectivity states and may ultimately facilitate integration of imaging with multimodal clinical information (84–86). Nevertheless, these architectures should currently be regarded as methodological opportunities rather than established tools in vestibular fMRI. Deep models typically contain substantially more trainable parameters than conventional machine-learning approaches and therefore place greater demands on sample size, data harmonization, and independent validation (59). These requirements are particularly challenging in vestibular neuroimaging, where available cohorts are generally small and multicenter datasets remain uncommon; under such conditions, simpler models with transparent feature selection and rigorous nested validation may provide more reliable and interpretable estimates than highly flexible deep architectures (60). Moreover, the core premise of supervised learning—that the diagnostic label is known as the “gold standard”—is itself uncertain in vertigo. Current diagnoses are based primarily on clinical symptoms rather than neurobiological evidence, meaning that a single diagnostic label may conceal markedly different patterns of brain network reorganization, while patients with different conditions may share similar network abnormalities. This uncertainty in the reference standard is a key reason why unsupervised learning is particularly valuable in vertigo: it allows candidate subtypes to emerge from the data without assuming that clinical labels correspond to homogeneous neurobiological entities.

### 4.4 The uncertainty of diagnostic labels and the value of unsupervised phenotyping

A further limitation of supervised learning is that the diagnostic label itself may not be a reliable “gold standard” in vertigo. Current diagnoses rely on clinical symptoms rather than neurobiological evidence, so a single label such as PPPD may conceal distinct patterns of brain-network reorganization, while different labels may share similar network abnormalities. This uncertainty motivates unsupervised approaches, which allow candidate subtypes to emerge without assuming that clinical labels define homogeneous neurobiological entities.

Unsupervised clustering of connectivity features, dynamic network states, or multimodal clinical–imaging variables could identify candidate subtypes. For example, vision-dependent vertigo characterized by high coupling in the visual network (63), a dominant phenotype of the vestibular-emotional integration network characterized by co-activation of the insula, secondary somatosensory cortex, and cerebellum (61), a subtype of chronic vestibular neuritis potentially characterized by delayed or impaired central compensatory processes (87), and multisensory instability driven by migraine susceptibility (88). However, caution is required when interpreting data-driven subtypes in PPPD. Unsupervised algorithms may group participants based on high variability unrelated to vestibular neurobiology, such as anxiety/depression severity, medication exposure, age, or center effects. Therefore, statistically stable clusters should not automatically be regarded as neurobiological subtypes. Before clinical translation, candidate subtypes must be shown to be independent of psychiatric burden and medication status, reproducible across sensitivity analyses and independent cohorts, and validated for prognostic value and treatment response. Experience from psychiatric neuroimaging provides an important cautionary precedent. A methodological replication of previously proposed resting-state connectivity-based depression biotypes found that neither the brain–symptom associations nor the resulting clusters remained statistically reliable under more rigorous testing (89). More recently, a registered report comparing six data-driven depression-subtyping approaches in 2,276 individuals with depression found almost no agreement in subtype assignment across methods (90). These findings illustrate that internally stable clustering solutions do not necessarily represent reproducible biological subtypes. Candidate vestibular phenotypes should therefore be evaluated across alternative analytical choices and replicated in independent cohorts before mechanistic or clinical meaning is assigned.

### 4.5 Explainability and biological validity

Explainability is particularly important if data-driven models are ultimately intended to support clinically meaningful interpretation. Explainable artificial intelligence (XAI) methods can help identify which imaging or clinical features contribute most strongly to model predictions (91, 92). SHapley Additive exPlanations (SHAP) can quantify the contribution of individual variables to patient-level predictions, with such attributions also being aggregated to characterize global feature importance (93, 94). For convolutional architectures, gradient-based visualization methods such as Grad-CAM may help localize spatial features contributing to classification (92, 95), whereas attention-based models can highlight patterns receiving greater computational weighting during prediction, although attention weights should not necessarily be interpreted as direct measures of feature importance (96, 97). However, explainability should not be equated with biological or causal validity (91). A feature receiving high importance in an AI model may reflect correlations, site effects, motion artifacts, medication exposure, or other confounding factors rather than disease-specific neurobiology (98–101). Similarly, attention weights should not automatically be interpreted as mechanistic explanations (97). XAI should therefore be combined with neurobiological plausibility, sensitivity analyses, independent replication, and longitudinal or statistical validation (78, 91). In vestibular fMRI, such integration may provide a more credible bridge between high-dimensional prediction and eventual clinical interpretation.

### 4.6 Current evidence gaps and sample-size considerations

Conventional machine learning, deep learning, and unsupervised phenotyping should be viewed as complementary rather than hierarchically ordered. Their suitability depends on sample size, data dimensionality, feature representation, clinical objectives, and validation design. The major limitation in vestibular fMRI is not the absence of sophisticated algorithms, but the scarcity of sufficiently large, harmonized, and independently validated datasets. Methodological complexity should not be prioritized over robustness, interpretability, and reproducibility. For inferential analyses screening large numbers of regions or connections, appropriate multiple-comparison procedures, such as false-discovery-rate or permutation-based correction, remain necessary; increasing sample size improves statistical power and stability but does not substitute for multiplicity control.

Sample-size requirements for reliable external validation cannot be reduced to a single threshold but critically depend on the phenotype’s underlying effect size and the sizes of both the training and independent validation samples (102). As an order-of-magnitude benchmark, recent large-scale neuroimaging simulations found that predictions with relatively large effects could achieve adequate power with training and external validation samples of a few hundred participants each, whereas medium or small effects could require hundreds to thousands of participants in both samples (102). These estimates were derived from broader brain–phenotype prediction studies and should not be interpreted as fixed sample-size thresholds for vestibular fMRI or unsupervised subtype discovery. Accordingly, small single-center vestibular fMRI cohorts should primarily be considered exploratory, whereas reproducible individual-level prediction requires larger samples and, critically, adequately powered external validation.

## 5 Advanced statistical modeling for longitudinal vestibular fMRI: current evidence gaps and future methodological directions

The artificial intelligence methods discussed in the previous section have been used primarily for cross-sectional classification and phenotyping in current vestibular fMRI research. Although machine-learning approaches can in principle model temporal and sequential data (84, 85), such applications remain largely underexplored in vestibular neuroimaging. In this review, mixed-effects, nonlinear, and trajectory models are therefore emphasized for addressing longitudinal questions such as within-subject change, nonlinear recovery, and heterogeneous compensatory trajectories. However, it is important to note that these advanced statistical models have rarely been applied directly to longitudinal vestibular fMRI to date. This section therefore presents them as priorities for future research, rather than as established analytical standards in this field. The brain network abnormalities of vestibular diseases are not static, and the stability of the brain network declines along a non-linear trajectory throughout the life cycle (14). Therefore, artificial intelligence cannot replace rigorous statistical modeling. AI is primarily suited to discrimination and pattern discovery, whereas longitudinal statistical models are designed to characterize within-subject change, nonlinear temporal patterns, and heterogeneous trajectories. In response to this demand, the following three advanced statistical models provide key tools for characterizing the dynamic evolution of vestibular disease brain networks.

### 5.1 Modeling hierarchical dependence in longitudinal and multicenter fMRI

In longitudinal or multicenter fMRI studies, repeated measurements are nested within participants, while participants may also be clustered within acquisition sites. Ignoring these dependencies can lead to misspecified covariance structures and biased statistical inference. Linear mixed-effects models (LMMs) address this problem by incorporating subject- and, where appropriate, site-level random effects. In vestibular fMRI, Ibitoye et al. (103) applied participant-specific random intercepts to account for repeated measurements, while Padova et al. (104) used random intercepts and slopes in longitudinal vestibular neuroimaging to model individual differences in baseline measures and rates of change. Longitudinal vestibular fMRI has also employed alternative repeated-measures approaches, such as sandwich-estimator models (105), reinforcing the need to account explicitly for within-subject dependence. In multicenter fMRI, substantial scanner- and site-related variability has been demonstrated, supporting either statistical harmonization or explicit modeling of site effects in hierarchical analyses (106, 107).

For longitudinal mixed-effects analyses, sample-size planning should be aligned with the prespecified primary model rather than based on a fixed participant threshold (108). Power depends on the anticipated effect size, number and timing of repeated scans, within-subject correlation, random-effect structure, site-level clustering (109), and expected attrition. Model-based or simulation-based power calculations should therefore be performed prospectively, particularly when random slopes or time-by-group interactions are of primary interest. Recruitment targets should additionally account for loss to follow-up and imaging data excluded because of motion or other quality-control failures (108).

### 5.2 Nonlinear models for temporal and clinical trajectories

The trajectories of central vestibular compensatory processes and their underlying brain networks over time are unlikely to follow a simple linear trend (7, 14). Modeling continuous variables only as linear predictors may therefore obscure nonlinear associations or inflection points. Spline-based approaches, including restricted cubic splines (RCS) and generalized additive models (GAMs), provide flexible means of evaluating such relationships without arbitrarily categorizing continuous variables. Large-scale fMRI evidence supports this rationale: Sun et al. (110) modeled functional connectome trajectories across 33,250 individuals using spline-based generalized additive models and identified distinct nonlinear lifespan patterns and inflection points. In the vestibular field, nonlinear associations have also been reported for physiological measures; for example, RCS analysis identified a threshold-dependent relationship between vestibulo-ocular reflex suppression and vestibular migraine risk (111). However, direct applications of these nonlinear models to vestibular fMRI remain limited. Their use should therefore be regarded as a promising approach for future studies examining nonlinear associations between network measures and age, disease duration, symptom burden, or treatment exposure, rather than as an established analytical standard.

### 5.3 Characterizing heterogeneous longitudinal trajectories

Longitudinal averaging may obscure meaningful inter-individual differences in brain changes. Latent trajectory approaches, including latent class growth analysis (LCGA) and growth mixture modeling (GMM), provide one possible framework for identifying subgroups with distinct patterns of change (112, 113). However, their direct application to longitudinal vestibular fMRI remains limited. These approaches should therefore currently be regarded as exploratory tools for testing whether reproducible patterns of network recovery or persistence emerge across repeated assessments, rather than as established analytical methods in vestibular fMRI (112, 113).

Latent trajectory models generally impose greater sample-size demands because the total cohort must support both reliable class enumeration and adequate representation of each latent subgroup. Simulation studies show that class recovery depends not only on total sample size but also on the number, prevalence, and separation of latent classes and on model specification (114). Studies evaluating samples from approximately 180 to more than 1,000 participants have shown substantially poorer class recovery under smaller or less favorable conditions, while even larger samples do not guarantee correct class enumeration when model assumptions are violated (115). Therefore, no fixed minimum sample size can be recommended for vestibular fMRI trajectory analysis. Monte Carlo simulation based on the anticipated number, size, separation, and shape of candidate trajectories should be used prospectively, and latent trajectory discovery should generally be reserved for substantially larger cohorts than those currently typical of single-center vestibular fMRI studies (114, 115).

In summary, the best analytical framework should combine machine learning with statistical modeling, rather than seeing it as a competitive approach. Machine learning can be used to screen high-dimensional imaging features and generate candidate biomarkers. It is important to note that a no-leakage strategy should be adopted during the machine learning screening phase; otherwise, the resulting “candidate biomarkers” may merely be products of overfitting and lack validation value (77). Subsequently, mixed-effects models and nonlinear models can be used to assess whether these biomarkers remain associated with clinically meaningful outcomes after controlling for confounding factors, clustering effects, and repeated measurements. The latent trajectory model can place the subtypes found by AI in a vertical framework to verify whether their evolution trajectories are truly separated. This strategy combines machine learning with statistical modeling: machine learning excels at identifying candidate patterns and features in high-dimensional data, whereas statistical modeling is suited to evaluating their longitudinal associations and clinical relevance. This design not only recognizes the unique advantages of the two methods, but also clarifies their respective boundaries and collaborative relationships. Figure 2 illustrates the unique advantages that each of these three types of advanced statistical models offers in characterizing vestibular brain networks, while Table 2 outlines the task boundaries for the two categories of methods—artificial intelligence and advanced statistical modeling—discussed in this review.

**Figure 2:**
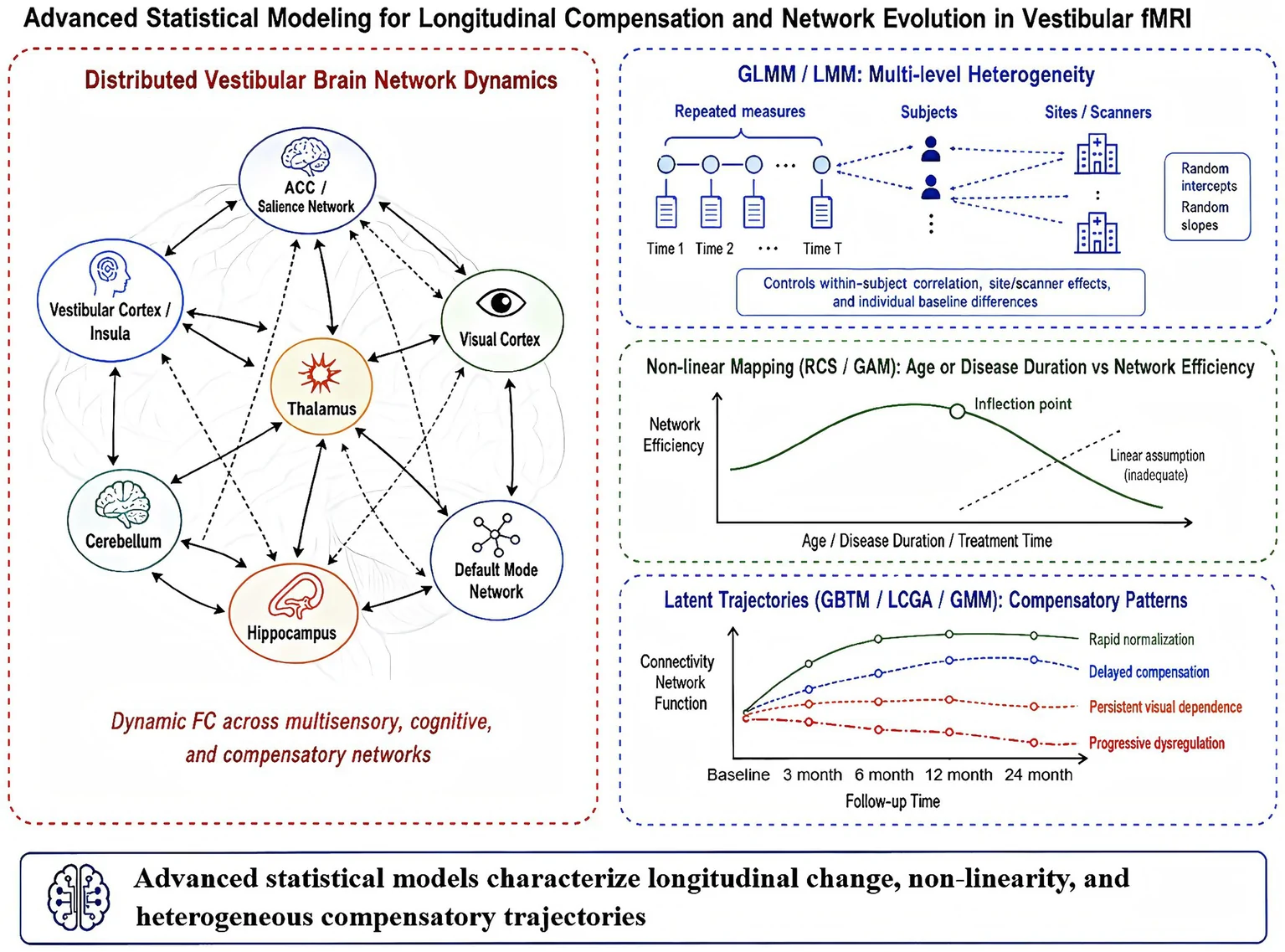
Conceptual statistical approaches for investigating longitudinal compensation and network evolution in vestibular fMRI.

**Table 2:** Comparison of core tasks and functional boundaries between artificial intelligence and advanced statistical modeling in vestibular fMRI research.

	Machine Learning/Artificial Intelligence	Advanced Statistical Modeling
Core mission	Classification and Subtype Identification	Explanation of longitudinal processes, characterization of nonlinear mechanisms
Input features	High-dimensional image features, adjacency matrices, graph theory metrics, dynamic network parameters	Longitudinal repeated-measure data, time-dependent variables, and multilevel covariates
Output	Category labels (diagnosis, subtype), Candidate diagnostic	Population average trajectory, individual rate of change, trajectory class, threshold/inflection point
Key advantages	Handling high-dimensional features and discovering hidden patterns	Control for confounding factors, model dynamic processes, and provide statistical inferences
Key limitations	Current vestibular fMRI applications are predominantly cross-sectional; longitudinal and temporal modeling remains underexplored, and causal interpretation requires appropriate study designs.	The model form and covariate structure must be specified in advance.

## 6 A proposed data-driven framework for mechanistic investigation and future clinical translation

Based on the aforementioned methodological advances, future vestibular fMRI studies may adopt the following six-step analytical framework. This framework integrates the entire process—from data collection and feature extraction to model development and clinical translation. Its core objective is not to replace statistical modeling with artificial intelligence, but rather to integrate the two into a single methodological framework where they operate in tandem (Figure 3).

**Figure 3:**
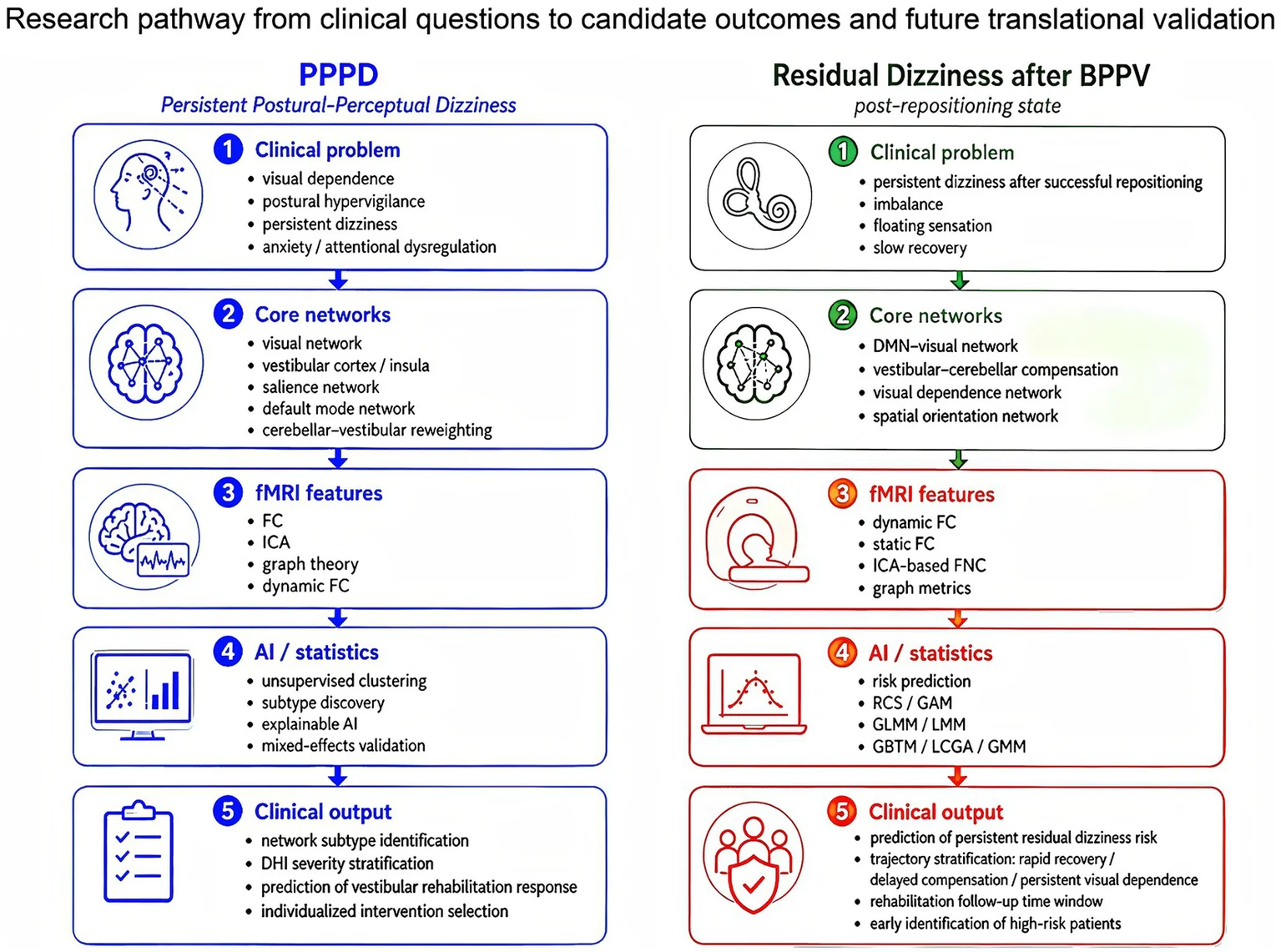
Proposed research pathway from clinical questions to candidate phenotypes, longitudinal validation, and future clinical translation.

Before implementing the six-step framework, Sample-size and power planning should precede model selection and should be tailored to the primary analytical objective. Requirements differ substantially between predictive machine learning, longitudinal mixed-effects modeling, and latent trajectory analysis; therefore, a single sample-size threshold should not be applied across the proposed framework.

Step 1: Clinical phenotype and confounder characterization. Participants should be classified according to internationally recognized diagnostic criteria, while vestibular subtype, symptom duration and severity, hearing status, vestibular function, migraine characteristics, and relevant comorbidities are systematically documented. Anxiety and depressive symptoms should be assessed using validated scales rather than recorded only as binary comorbid diagnoses. Medication exposure should likewise be characterized by drug class, dose, treatment duration, recent treatment changes, and timing relative to MRI acquisition. The potential role of these variables as confounders, effect modifiers, or phenotype-defining features should be prespecified according to the research question.

Step 2: Coordinate multi-center imaging acquisition and preprocessing. Standardize MRI scanning parameters and preprocessing workflows across all study sites, including head motion correction, temporal alignment, subtraction of noise signals, spatial normalization, quality control, and recording of scanner/site codes. Vestibular-specific issues related to head motion, scan tolerability, medication exposure, and task standardization should additionally be addressed as described in Section 3.

Step 3: fMRI feature extraction. The network features with biological explanatory power were extracted from the preprocessed images, including regional activity index, static and dynamic functional connectivity matrix, graph theory topological parameters and network-level generalization index. These features constitute the common input space of artificial intelligence model and statistical model.

Step 4: Development of predictive and classification models. Before developing predictive and classification models, the adequacy of the sample size and the feasibility of retaining an independent validation set should be assessed. Machine learning-based diagnostic classification or subtype identification models should then be constructed using leak-free feature selection, nested cross-validation, and calibration assessment; where feasible, external independent validation should be incorporated to evaluate the model’s generalization ability. In addition, model development should examine whether predictive performance or identified subtypes are driven disproportionately by psychiatric symptom severity, medication exposure, or other non-vestibular variables. Sensitivity analyses with and without these variables, as well as stratified analyses where feasible, can help evaluate the robustness of imaging-derived patterns.

Step 5: Statistical modeling of longitudinal evolution and treatment response. For the features extracted in step 3 and the potential subtypes identified in step 4, mixed-effect models, nonlinear models, and latent trajectory models should be applied to evaluate longitudinal network evolution, treatment response, and central compensation. Importantly, the longitudinal design should be supported by model-specific power calculations that account for repeated assessments, attrition, and, where latent trajectories are investigated, the expected size of the smallest subgroup. Furthermore, when interpreting network evolution, changes in anxiety, depression, and medication exposure should be considered; when measurements are available at repeated visits, these factors may be modeled as time-varying covariates or examined in sensitivity and mediation-oriented analyses, depending on the causal question.

Step 6: Biological interpretation and future clinical validation. Candidate imaging-derived biomarkers or data-driven phenotypes should first be interpreted within a biological and clinical research framework rather than directly applied for individual treatment decisions. Associations with symptoms, functional outcomes, and treatment response should be evaluated prospectively. Before fMRI-plus-AI approaches can inform patient-level clinical decisions, their reliability, generalizability, and incremental value beyond conventional clinical assessment require validation in large, independent cohorts.

### 6.1 Two priority research scenarios across the vestibular hierarchy

The two priority scenarios are positioned at different levels along a peripheral-to-central hierarchy of vestibular dysfunction, rather than as two parallel applications of the same analytical problem. Residual dizziness following BPPV repositioning represents the more peripheral end of this hierarchy, providing a model for investigating longitudinal central readaptation after correction of a peripheral vestibular disturbance. PPPD, by contrast, represents the higher-order end, characterized by persistent alterations in perceptual, attentional, and multisensory network organization. This conceptual distinction motivates the different but overlapping analytical strategies proposed for the two scenarios. This conceptual distinction motivates the different but overlapping analytical strategies proposed for the two scenarios.

Scenario 1: Identification of PPPD subtypes. Steps 1–2 involve the standardized collection of clinical phenotypes and multicenter fMRI data; Step 3 involves extracting static and dynamic functional connectivity, graph theory metrics, and cross-network coupling features; Step 4 uses unsupervised clustering to identify candidate internal subgroups—such as a vision-dependent subtype and an emotion-limbic system-dominant subtype—that may improve understanding of symptom heterogeneity and generate hypotheses for future treatment stratification; Step 5 uses longitudinal follow-up to examine whether these candidate subtypes show differential treatment response, thereby providing preliminary evidence for their potential clinical relevance; Step 6: Translate these findings into research-informed hypotheses for future prospective validation, rather than immediate clinical decision-making.

Scenario 2: Longitudinal characterization of residual dizziness following BPPV repositioning. Current fMRI evidence for BPPV residual dizziness remains limited. Cross-sectional studies have identified altered functional connectivity involving visual, default-mode, and vestibular-related networks after successful repositioning (59), while a recent randomized longitudinal rs-fMRI study demonstrated functional-connectivity changes following 4 weeks of vestibular rehabilitation (48). However, these findings do not establish whether patients follow distinct spontaneous recovery trajectories. Within the proposed framework, repeated clinical and fMRI assessments could therefore be used to test whether candidate patterns such as rapid normalization, delayed recovery, or persistent visual–vestibular abnormalities are reproducible. These trajectories should be regarded as testable hypotheses rather than established fMRI phenotypes, and their associations with symptom recovery or rehabilitation response require prospective validation.

## 7 Discussion

The central argument of this review is that, in fMRI studies of vertigo, artificial intelligence and advanced statistical modeling form a complementary methodological pipeline rather than competing analytical strategies. This position is not a simple compromise; rather, it is based on the fundamental difference in the kinds of questions the two approaches can answer. Artificial intelligence, including supervised classification and unsupervised clustering, can identify multivariate brain-network patterns and generate candidate diagnostic or subtype assignments within research datasets, thereby characterizing network states at a given time point. Advanced statistical modeling, including mixed-effects models, nonlinear models, and latent trajectory models, addresses how these network states evolve over time, thereby explaining the dynamic processes of brain networks. The former provides classification and hypothesis generation, while the latter provides verification and mechanistic inference. Each has a distinct role, and both are necessary.

### 7.1 Application limits: what these two methods can and cannot do

The establishment of this complementary relationship stems precisely from a clear understanding of the limitations of each approach. Artificial intelligence can identify candidate biomarkers from high-dimensional fMRI features and generate individual-level predictions or candidate subtype assignments within research datasets. However, such outputs should not be interpreted as clinically valid patient-level classifications unless their reliability, generalizability, and incremental clinical value have been established prospectively. Especially in the field of vertigo, when the diagnostic label itself may be based on symptoms rather than neurobiological mechanisms, unsupervised clustering is expected to break through the existing classification framework and discover new cross-diagnostic network subtypes. Current AI applications in vestibular fMRI have largely focused on cross-sectional classification and phenotyping rather than longitudinal trajectory modeling. Although machine-learning approaches can model temporal data, longitudinal statistical models provide established frameworks for repeated-measures analysis, covariate adjustment, and characterization of within-subject change over time. Importantly, neither AI nor conventional longitudinal statistical models can establish causality without an appropriate causal study design. Without subsequent statistical validation, the subtype hypotheses it generates may merely describe data structures rather than accurately reflect biological reality. Advanced statistical modeling can deal with the hierarchical structure in longitudinal repeated measurements, control multi-center heterogeneity, characterize the nonlinear characteristics of compensatory trajectories, and identify potential heterogeneous evolution patterns in the population. These capabilities effectively address the limitations of artificial intelligence. However, statistical modeling is not a panacea; it relies on predefined model forms and covariate structures, requires sufficient sample sizes and longitudinal follow-up data, and is less flexible than artificial intelligence when it comes to feature selection and high-dimensional pattern recognition. In short, AI excels at “identifying features” and “formulating hypotheses,” while statistical modeling excels at “testing hypotheses” and “inferring mechanisms.” Integrating these two approaches into a single methodological framework is precisely the integrated approach advocated in this review.

### 7.2 Why this division of labor? Insights from fMRI network phenotypes of vertigo

The necessity of this division of labor is not merely a methodological deduction, but stems from empirical findings in fMRI studies of vertigo. As described in Section 2.3, various vestibular disorders—including VM, PPPD, MD, CUVP, and BPPV—exhibit overlapping network phenotypes across diagnoses in terms of default mode networks, visual networks, salience networks, and whole-brain topological efficiency, while significant individual variations are observed within each diagnostic group.

This finding directly points to two conclusions: First, since network phenotypes do not fully align with clinical diagnostic labels, relying solely on supervised classification to “distinguish known diseases” is insufficient. It is necessary to explore, within an unsupervised framework, whether more reasonable network subtypes exist—and this is precisely where the value of AI lies. Second, since these network anomalies evolve dynamically—with compensatory mechanisms potentially progressing rapidly before leveling off, and visual reliance gradually strengthening—a static assessment based on a single point in time cannot answer questions such as “Is this anomaly temporary or persistent?” and “How will it evolve?” This is precisely where longitudinal statistical modeling comes into play. Therefore, the proposal of the complementary framework is essentially a methodological response to the dynamic nature of the vertigo brain network.

### 7.3 Current gaps: evidence bottlenecks for methodological implementation

Although the above framework provides a plausible methodological strategy, its empirical implementation in longitudinal vestibular fMRI remains limited. Most existing studies are cross-sectional, while longitudinal studies remain relatively uncommon and frequently emphasize clinical outcomes rather than repeated brain-network measures. Mixed-effects analyses of resting-state functional connectivity or graph-theoretical measures remain rarely reported, and nonlinear approaches have only occasionally been applied to characterize recovery-related changes. Direct applications of latent trajectory models to longitudinal vestibular fMRI appear particularly sparse. These methodological gaps are not isolated from one another; rather, they collectively point to a structural issue: vestibular fMRI research remains dominated by descriptive population-level mapping, with limited data and methodological resources for longitudinal characterization of network dynamics. Importantly, the current limitation is not only the scarcity of longitudinal datasets but also their scale: cohorts adequate for conventional group-level analyses may still be insufficient for stable machine-learning phenotyping or latent trajectory discovery. Future multicenter cohort development should therefore be driven by prespecified analytical objectives and model-specific power calculations rather than by convenience-based recruitment alone.

### 7.4 Priorities for methodological validation and future translation

The immediate priority for data-driven vestibular fMRI research is methodological validation rather than clinical deployment. Future studies should establish whether candidate imaging features, network phenotypes, and longitudinal patterns are reproducible in sufficiently large, longitudinal, and multicenter cohorts. Prospective sample-size planning, harmonized acquisition and preprocessing, transparent model development, and independent or external-site validation should therefore be incorporated at the study-design stage.

For AI-based phenotyping, emphasis should be placed on reproducibility rather than algorithmic complexity. Candidate subtypes should be tested for sensitivity to feature selection, preprocessing, psychiatric symptoms, medication exposure, and site effects, while proposed longitudinal patterns should be evaluated using repeated imaging rather than inferred from cross-sectional associations. The framework proposed here should therefore be viewed as a research strategy for hypothesis generation and validation. Individual-level clinical application would require substantially stronger evidence, including prospective replication and demonstrated incremental value beyond established clinical assessment.

### 7.5 Limitations of the current evidence and review

Several limitations should be considered. First, direct empirical evidence combining vestibular fMRI with artificial intelligence and advanced longitudinal modeling remains limited. Most available vestibular fMRI studies are cross-sectional and group-based, while many AI studies in vestibular medicine rely on non-fMRI data. Some methodological recommendations in this review are therefore informed by related neuroimaging, psychiatric, clinical, or statistical research and should be regarded as methodological precedents rather than direct validation in vestibular populations.

Second, the current evidence base is constrained by small samples, predominantly single-center designs, heterogeneous acquisition and preprocessing procedures, and limited external validation. Technical and biological factors—including head motion, physiological noise, scanner/site effects, psychiatric symptom burden, and medication exposure—may also influence BOLD-derived measures and data-driven phenotyping. These issues reinforce the need for prospectively powered multicenter studies, harmonized procedures, and prespecified sensitivity and validation analyses.

Third, the candidate phenotypes and longitudinal patterns discussed in this review remain hypothesis-generating. Proposed PPPD-related network phenotypes and possible recovery patterns following BPPV repositioning have not been established as reproducible neurobiological entities and should not currently be interpreted as clinically actionable subtypes or trajectories.

Finally, this article was designed as a structured narrative methodological review rather than a systematic review or quantitative meta-analysis. The literature was selected to address predefined methodological questions and priority research scenarios rather than to provide exhaustive coverage of all vestibular studies. Given the heterogeneity of study populations, imaging protocols, analytical pipelines, and outcome definitions, quantitative pooling of neuroimaging effects or model performance would not currently be appropriate. Future systematic reviews and meta-analyses may become more informative as the evidence base becomes larger and methodologically more comparable.

Overall, the principal priority is not greater algorithmic complexity, but the development of sufficiently large, longitudinal, harmonized, and independently validated vestibular fMRI datasets capable of testing whether the proposed phenotypes and longitudinal patterns are reproducible.

## 8 Conclusion

fMRI, artificial intelligence, and advanced statistical modeling provide important methodological tools for vestibular neuroimaging. Their immediate value lies in addressing specific research questions that are difficult to resolve using conventional assessments. In this review, PPPD and residual dizziness following BPPV repositioning are proposed as two priority research scenarios: the former for investigating heterogeneity in brain-network phenotypes and treatment response, and the latter for characterizing incomplete central compensation and its longitudinal recovery. AI may support high-dimensional pattern discovery and candidate subtype identification, whereas advanced statistical models can evaluate the longitudinal clinical relevance of these candidate features and phenotypes. Ultimately, this framework should currently be viewed as a research roadmap for identifying and validating neurobiological patterns associated with vestibular symptom heterogeneity. Whether such patterns can provide clinically actionable information at the individual patient level remains an important question requiring prospective validation, multicenter replication, and comparison with established clinical assessment approaches.
